# Magnetically Diluted Dy^3+^ and Yb^3+^ Squarates Showing Relaxation Tuning and Matrix Dependence

**DOI:** 10.3390/molecules30020356

**Published:** 2025-01-16

**Authors:** Rina Takano, Takayuki Ishida

**Affiliations:** Department of Engineering Science, The University of Electro-Communications, Chofu 182-8585, Tokyo, Japan; takano@ttf.pc.uec.ac.jp

**Keywords:** rare earth metal, lanthanide, single-ion magnet, magnetization reversal, relaxation mechanism

## Abstract

A new compound [Y_2_(sq)_3_(H_2_O)_4_] (Y-sq; sq = squarate (C_4_O_4_^2–^)) was prepared and structurally characterized. Since the RE-sq family (RE = Y, Dy, Yb, Lu) gave isostructural crystals, the objective of this study is to explore the effects of diamagnetic dilution on the SIM behavior through systematic investigation and comparison of diamagnetically diluted and undiluted forms. The 1%-Diluted Dy compounds, Dy@Y-sq and Dy@Lu-sq, showed AC magnetic susceptibility peaks without any DC bias field (*H*_DC_), whereas undiluted Dy-sq showed no AC out-of-phase response under the same conditions. The Orbach and Raman mechanisms are assumed in the Arrhenius plots, giving *U*_eff_/*k*_B_ = 139(5) and 135(8) K for Dy@Y-sq and Dy@Lu-sq, respectively, at *H*_DC_ = 0 Oe. In contrast, Yb@Y-sq and Yb@Lu-sq behaved different; Yb@Y-sq can be regarded as a field-induced SIM because AC out-of-phase response was recorded only when *H*_DC_ was present. On the other hand, Yb@Lu-sq showed a relaxation independent from temperature around 2 K at *H*_DC_ = 0 Oe, possibly ascribed to a quantum-tunneling-magnetization mechanism. Applying *H*_DC_ = 400 Oe afforded *U*_eff_ = 61.2(14) and 62.5(16) K for Yb@Y-sq and Yb@Lu-sq, respectively. The Y/Lu matrix dependence may be related to spin–phonon coupling. The dilution technique is a facile and versatile tool for modification of SIM characteristics.

## 1. Introduction

Single-ion magnets (SIMs) are a class of materials that have garnered significant interest due to their potential applications in quantum computing, high-density data storage, and molecular spintronics. These materials exhibit a slow relaxation of magnetization at the molecular level, a property that is predominantly observed in lanthanide-based complexes due to their large magnetic anisotropy and high-spin ground states [1,2,3,4]. Among the lanthanides, dysprosium (Dy) is particularly noteworthy for its distinct magnetic properties; Dy^3+^ ions, with their high magnetic moment and strong spin–orbit coupling, have been extensively studied in SIMs [5,6,7,8], demonstrating significant barriers to magnetization reversal. The current record effective energy barrier *U*_eff_ is 1687 cm^−1^ for [Dy(OAd)_2_(18-crown-6)]I_3_ [8]. Ytterbium (Yb) ions, on the other hand, offer an interesting contrast due to their smallest magnetic moment (*S* = 1/2), and, accordingly, Yb-based SIMs remain relatively underexplored [9,10,11,12]. The prescription toward high-performance Yb SIMs is not sufficiently known. The study of these ions in various coordination environments provides invaluable insights into the design principles of SIMs.

A significant aspect of our research involves the examination of diamagnetic dilution by using the isostructural crystalline solid family. Diamagnetic dilution, wherein a fraction of the magnetic ions is replaced with non-magnetic ions, is a common strategy to probe the relaxation dynamics in SIMs [13,14,15,16]. It removes dipolar, exchange, and other intermolecular interaction, leading to reduction in possible quantum tunneling of magnetization (QTM). Our diamagnetic diluted samples exhibited obviously different alternating current (AC) magnetic susceptibility behaviors compared to their undiluted counterparts. This observation may afford information on the presence of an additional relaxation mechanism that warrants further exploration. The relaxation of magnetization in SIMs can be influenced by various mechanisms, including Raman processes and direct relaxation pathways as well as QTM [17,18,19]. The dilution of magnetic ions can either enhance or obscure these mechanisms, making it challenging to isolate the dominant relaxation pathway. Previous studies have shown that magnetic dilution can sometimes reveal hidden relaxation processes or modify the existing ones.

**Scheme 1:**
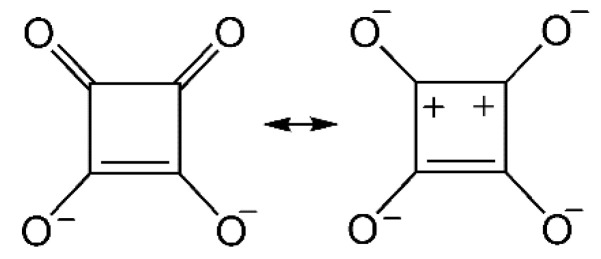
Canonical structures of squarate (sq).

Recent advancements have explored the use of polymeric complexes as hosts for these lanthanide ions [20,21,22]. A dianion of 3,4-dihydroxycyclobut-3-ene-1,2-dione or simply squarate (abbreviated as sq) has 2π-aromaticity in its dicationic ring [23], thus enhancing anionic charge on four oxygen atoms (Scheme 1). As a result, sq can serve as a versatile O-donor [24,25,26,27,28]. The combination of RE ions and squaric acid offers a platform for designing novel SIMs with tailored magnetic properties. In fact, a double-layered two-dimensional (2D) polynuclear complex [Tb_2_(sq)_3_(H_2_O)_8_]*_n_* was reported as a candidate for a potential luminescent magnet [29]. We synthesized and characterized analogous complexes containing either Dy or Yb ions [12]. The choice of single-component lanthanide ion in these frameworks allows for a detailed investigation of their intrinsic physical properties.

The objective of this study is to elucidate the relaxation dynamics in 2D polymeric complexes of Dy and Yb ions under the influence of diamagnetic dilution and applying magnetic field. Diamagnetic Y^3+^ and Lu^3+^ are chosen as matrix ions for dilution. By comparing the AC magnetic susceptibility of single-component lanthanide complexes with their diluted derivatives, we aim to identify the contributing relaxation mechanisms and understand their dependence on the magnetic environment.

## 2. Results

A new compound Y-sq was prepared according to the method generalized for related RE-sq [29]. In the present study, we investigated magnetically diluted compounds from Dy-sq and Yb-sq into a Y-sq diamagnetic host, taking advantage of the isostructural nature among them. Preliminary results on the dilution experiments using Lu-sq are already described [12]. Diluted samples, Dy@Y-sq, Dy@Lu-sq, Yb@Y-sq, and Yb@Lu-sq were prepared in a manner similar to that of undiluted RE-sq samples. The starting lanthanide acetate salts were uniformly mixed with a molar ratio of 1/99, before the complexation reaction. The target materials crystallized from the corresponding aqueous solution of the mixture. The elemental analysis supported the composition, though these content values are practically the same as those of the matrix Y- and Lu-sq compounds. The FT-IR data of the diluted samples were confirmed to be identical to those of host RE-sq.

Before detailed study, the direct-current (DC) magnetic properties were measured to ensure the dopant-RE/matrix-RE molar ratios. In fact, the ratios were found to vary from 0.8 to 1.9% for the present diluted materials. These contents seem to be satisfactory within a limited accuracy because of the relatively small paramagnetic entity against the diamagnetism (Figures S1 and S2 in Supplementary Materials).

**Figure 1:**
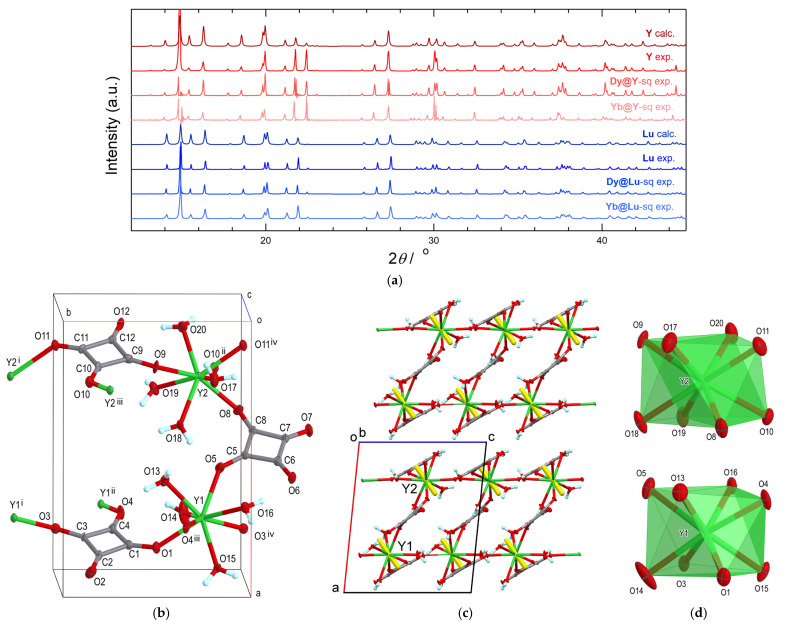
(**a**) Experimental and calculated powder XRD profiles for RE-sq. (**b**) X-ray crystal structure of [Y_2_(sq)_3_(H_2_O)_8_]*_n_*. Thermal ellipsoids are drawn at the 50% probability level. Symmetry operation code: i: *x*, *y* + 1, *z*; ii: *x*, –*y* + 1, *z* + 1/2; iii: *x*, –*y* + 1, *z*–1/2; iv: *x*, *y*–1, *z*. Color code: C: gray; H: turquoise; O: red; Y: green. (**c**) Molecular arrangement viewed along the *b* axis. Yellow thick lines stand for the direction between two basal plane centroids in a YO_8_ square antiprism. (**d**) Coordination polyhedra for the Y^3+^ ions in [Y_2_(sq)_3_(H_2_O)_8_]*_n_*.

The phase purity was confirmed by means of powder X-ray diffraction study (Figure 1a). Since the various crystal packing motives are known from RE and squarate systems [28,29,30,31,32], selective preparation methods are required for a particular crystalline motif. Eventually, we explored the selective preparation for the RE-sq type from lanthanide acetate hydrates as starting materials [12,29]. The PXRD profiles of Dy@Lu-sq, Dy@Y-sq, Yb@Lu-sq, and Yb@Y-sq were found to be practically identical to that of Y-sq and Lu-sq. From these crystal motives, spectroscopic and emission properties [29,31,33,34,35] and magnetic properties [12,29,31,36] have extensively been investigated for a few decades.

The crystal structures of 2D [RE_2_(sq)_3_(H_2_O)_8_]*_n_* (RE-sq; RE = Eu, Gd, Tb, Dy, Ho, Er, Tm, Yb, Lu) have systematically been characterized [12,29], and the Y counterpart, Y-sq, as a new compound was found to possess an isomorphic structure (Figure 1b–d). Only the essence will be noted here. It consists of a bilayer structure, where every sq bridges RE ions (Figure 1b). Squarate C1-C2-C3-C4 binds three RE1 ions with the O1, O3, and O4 coordination bonds, and an RE1/sq-sheet is formed parallel to the *bc* plane. Quite similarly, RE2 cations and C9-C10-C11-C12 sq anions construct an RE2/sq-sheet with the O9, O10, and O11 coordination bonds. Another sq interconnects RE1 and RE2 ions with the O5 and O8 donor atoms, thus constructing a 2D polynuclear sandwich (Figure 1c). The SHAPE analysis [37] reported that the YO_8_ coordination structures are best described as a square antiprism (SAPR-8) with the CShM values of 0.398 and 0.333 for the Y1 and Y2, respectively (Figure 1d). These values are comparable to those of RE-sq; CShM = 0.290 − 0.492 for RE = Eu − Lu with respect to SAPR-8 [12,29]. The importance of π-π interaction has already been pointed out for Eu-sq [30], because the sq rings are stacked along the *a* axis (Figure 1b). Furthermore, the presence of hydrogen bonds has also been clarified, exemplified with Tb-sq [29].

The O_4_ basal squares in the SAPRs are located almost parallel, as indicated with dihedral angles 3.25 and 1.19° for the Y1 and Y2 SAPRs, respectively. In addition, the two SAPRs are also located practically parallel; the approximate fourfold (*S*_4_) principal axis directions are aligned within 1.5° (Figure 1d). Although the two Y ions are crystallographically independent, the coordination circumstances are quite similar to each other. The local anisotropy axis may be parallel in each RE-sq material, when RE is magnetic. Such geometry is beneficial for slow magnetization reorientation [38,39].

**Figure 2:**
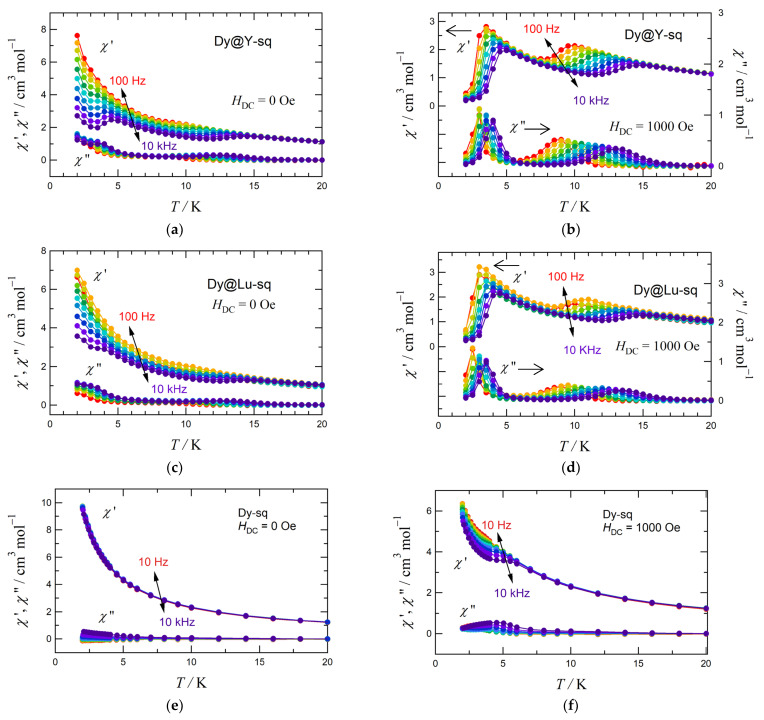
(**a**) AC magnetic susceptibilities, in-phase *χ*_AC_′ and out-of-phase *χ*_AC_″, for 1% Dy-sq diluted in a Y-sq matrix (Dy@Y-sq), measured without any DC bias field. The susceptibilities are converted per the undiluted composition formula [RE_2_(sq)_3_(H_2_O)_8_]. Lines are drawn only for a guide to the eye. (**b**) For 1%-diluted Dy@Y-sq, measured at a DC field of 1000 Oe. (**c**) AC magnetic susceptibilities for 1%-diluted Dy@Lu-sq, measured without any DC field. (**d**) For 1%-diluted Dy@Lu-sq, measured at a DC field of 1000 Oe. (**e**) AC magnetic susceptibilities for undiluted Dy-sq [12], measured at the DC fields of 0 Oe and (**f**) at 1000 Oe.

Figure 2 displays the in-phase and out-of-phase portions of the AC magnetic susceptibilities (*χ*_AC_′ and *χ*_AC_″, respectively) for the diluted Dy@Y-sq samples, measured at applied DC fields (*H*_DC_’s) of 0 and 1000 Oe. The DC field was chosen from that of the undiluted measurements on Dy-sq for the sake of convenience of comparison (for details, see the previous work [12]). A small *χ*_AC_″ was recorded at *H*_DC_ = 0 Oe (Figure 2a,c,e). Possible QTM is suppressed with a field bias [17]. In Figure 2b,d, two distinct *χ*_AC_″ peaks are clearly obtained around 2–5 and 8–13 K at *H*_DC_ = 1000 Oe. The low-temperature *χ*_AC_″ peaks are sharper than those observed in Dy-sq, and additional peaks not seen in Dy-sq are present around high-temperature region [12]. The high-temperature signals seem to be intrinsically due to the Orbach-type relaxation, while the low-temperature peaks might be artifact as a result of the suppression of QTM. The field dependence measurements were also performed (Figure S5, Supplementary Materials), showing that the low-temperature secondary peaks decreased at *H*_DC_ = 1000 Oe. In contrast, the high-temperature peak changed only slightly. Therefore, the origins of the two peaks are clearly different and must be ascribed both to Dy ions. As Figure 2b,d shows, the AC magnetic susceptibilities for Dy@Lu-sq samples are quite similar to those for Dy@Y-sq.

Note that Dy@Y-sq and Dy@Lu-sq showed an appreciable relaxation at zero field, indicating that they intrinsically are SIMs and guaranteeing coercivity and spontaneous magnetization. As for the undiluted Dy-sq, no meaningful relaxation was recorded [12]. The effect of dilution is dramatic. Such a dilution effect showing double peaks (Figure 2a–d) seems to be similar to that of applying bias field to undiluted sample (Figure S6). The high-temperature *χ*_AC_″ peaks were recorded for Dy-sq at *H*_DC_ = 3000 Oe. This behavior was observed for diluted samples as well, but a much weaker magnetic field was required.

**Figure 3:**
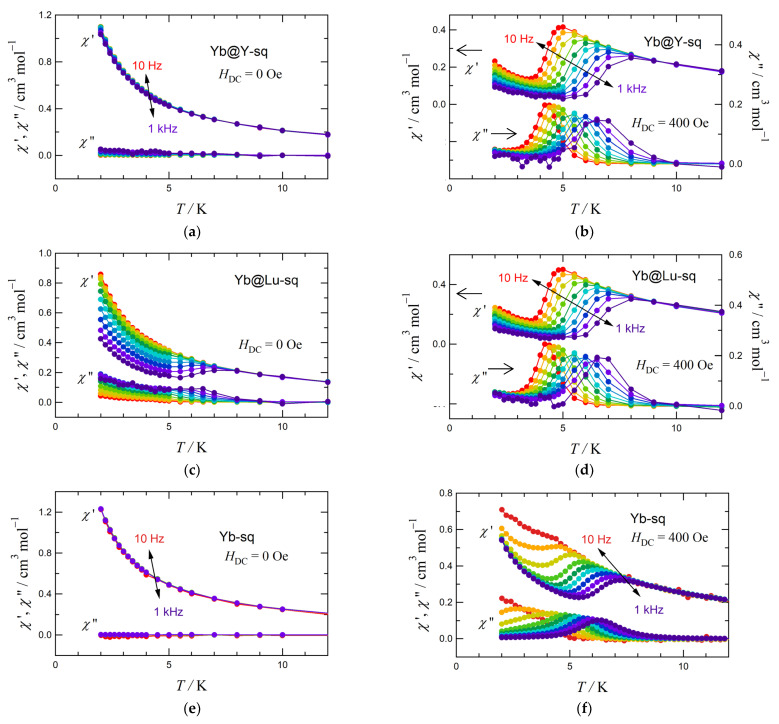
(**a**) AC magnetic susceptibilities, in-phase *χ*_AC_′ and out-of-phase *χ*_AC_″, for 1% Yb-sq diluted in a Y-sq matrix (Yb@Y-sq), measured without any DC bias field. The susceptibilities are converted per the undiluted composition formula [RE_2_(sq)_3_(H_2_O)_8_]. Lines are drawn only for a guide to the eye. (**b**) For 1%-diluted Yb@Y-sq, measured at a DC field of 400 Oe. (**c**) AC magnetic susceptibilities for 1%-diluted Yb@Lu-sq, measured without any DC field. (**d**) For 1%-diluted Yb@Lu-sq, measured at a DC field of 400 Oe. (**e**) AC magnetic susceptibilities for undiluted Yb-sq [12], measured at the DC fields of 0 Oe and (**f**) at 400 Oe.

Figure 3 shows the *χ*_AC_′ and *χ*_AC_″ vs. *T* plots for Yb derivatives diluted with Y or Lu ions. Different magnetic behaviors were observed, depending on diamagnetic diluting ions at *H*_DC_ = 0 Oe (Figure 3a,c,e). The Yb@Y-sq sample showed no frequency dependence. In sharp contrast, Yb@Lu-sq exhibited an increase in *χ*_AC_″ in a low-temperature region. The bias field was chosen to be the same as in the previous work [12]. The Yb@Y-sq and Yb@Lu-sq samples exhibited similar frequency dependence around 4–7 K to each other at *H*_DC_ = 400 Oe (Figure 3b,d), and their behavior partly resembled that of Yb-sq (Figure 3f). Namely, one peak appeared in common, but low-temperature behavior was different; in diluted samples of Yb@Y-sq and Yb@Lu-sq, the relaxation was sufficiently suppressed, while undiluted Yb-sq still showed a large susceptibility upsurge.

**Figure 4:**
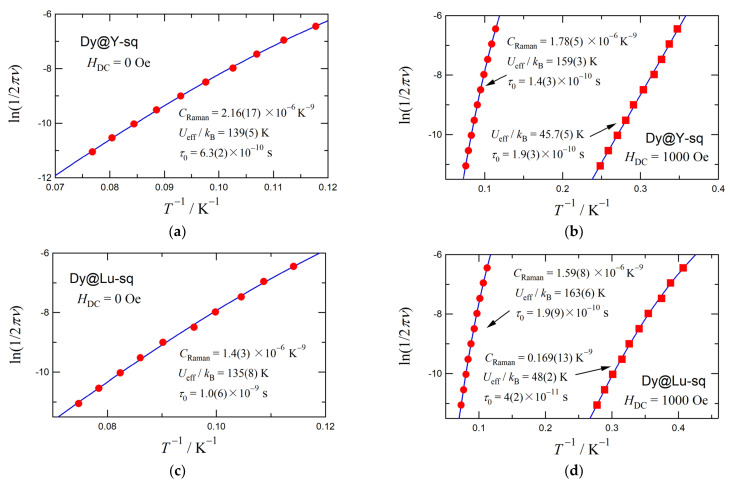
The Arrhenius plot for Dy@Y-sq at *H*_DC_ = 0 Oe (**a**) and at *H*_DC_ = 1000 Oe (**b**), and Dy@Lu-sq at *H*_DC_ = 0 Oe (**c**) and at *H*_DC_ = 1000 Oe (**d**). The data of (**a**–**d**) were given from Figure 2a, b, c, and d, respectively. Solid lines are drawn from the parameter optimization. For details, see the text.

The Arrhenius plots for Dy@Y-sq and Dy@Lu-sq are shown in Figure 4, based on the frequency dependence of the *χ*_AC_″ peak. The effective activation energy (*U*_eff_) for the magnetization reversal and pre-exponential factor (*τ*_0_) were determined according to Equation (1) that includes the Raman relaxation process [17,18,19,40];

(1) $$\ln\left(1/2\pi \nu \right)=\ln\left(\tau_{0}\right)+U_{eff}/k_{B}T+CT^{n_{Raman}}$$

The exponential temperature factor *n*_Raman_ is 9 because Dy^3+^ is a Kramers ion [18]. The fitting results of the Arrhenius plots for the high-temperature side were *U*_eff_/*k*_B_ = 139(5) K and *τ*_0_ = 6.3(2) × 10^−10^ s for Dy@Y-sq, and *U*_eff_/*k*_B_ = 135(8) K and *τ*_0_ = 1.0(6) × 10^−9^ s for Dy@Lu-sq at *H*_DC_ = 0 Oe. The *U*_eff_/*k*_B_ values for high temperature region were 159(3) K for Dy@Y-sq and 163(6) K for Dy@Lu-sq, which are larger than those obtained before applying the bias field, as the tunneling pathway was suppressed. The Arrhenius plots in a low-temperature region were fit with a line considering only the Orbach process for Dy@Y-sq, and the parameters were optimized as *U*_eff_/*k*_B_ = 45.7(5) K and *τ*_0_ = 1.9(3) × 10^−10^ s. The Arrhenius plots for Dy@Lu-sq showed a slightly convex curve, and the parameters were determined as *U*_eff_/*k*_B_ = 48(2) K and *τ*_0_ = 4(2) × 10^−11^ s, assuming that the Raman process as well as the Orbach process are included. No magnetic hysteresis was recorded in a usual timescale of the SQUID apparatus. The *M-H* curves and field-cooled magnetization (FCM), zero-field-cooled magnetization (ZFCM), and remnant magnetization (RM) on Dy@Y-sq and Dy@Lu-sq are shown in Figures S2c, S3, and S4.

**Figure 5:**
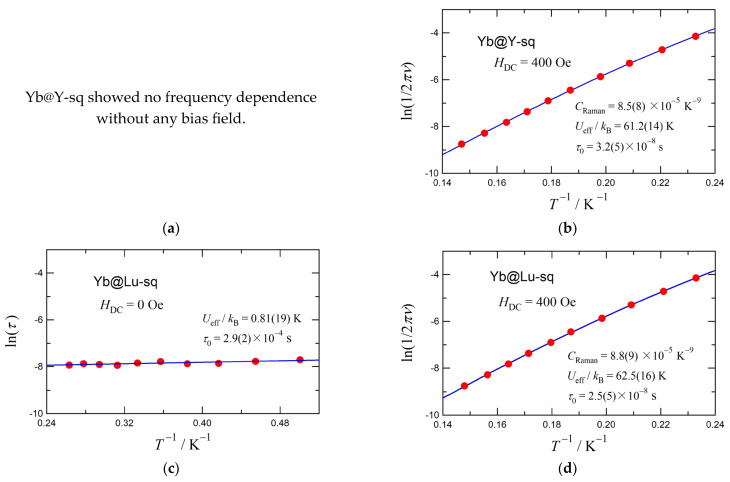
The Arrhenius plot for Yb@Y-sq at *H*_DC_ = 0 Oe (**a**) and at *H*_DC_ = 400 Oe (**b**), and Yb@Lu-sq at *H*_DC_ = 0 Oe (**c**) and at *H*_DC_ = 400 Oe (**d**). The data of (**a**–**d**) were given from Figure 3a, b, c, and d, respectively. Solid lines are drawn from the parameter optimization. For details, see the text.

The Arrhenius analysis for Yb@Y-sq and Yb@Lu-sq show no significant differences to each other when magnetic field is applied (Figure 5). The optimized parameters for these materials are *U*_eff_/*k*_B_ = 61.2(14) K and *τ*_0_ = 3.2(5) × 10^−8^ s for Yb@Y-sq (Figure 5b), and *U*_eff_/*k*_B_ = 62.5(16) K and *τ*_0_ = 2.5(5) × 10^−8^ s for Yb@Lu-sq (Figure 5d). The AC magnetic susceptibility of Yb@Lu-sq with no bias field hardly allows us to draw the Arrhenius plot because there were no *χ*_AC_″ peak (Figure 3c). The generalized Debye model was applied to obtain the Arrhenius plots (Table S1). The fit data well coincides with the experimental data (Figure S7). After the Debye model treatment, the Arrhenius parameters were optimized as *U*_eff_/*k*_B_ = 0.81(19) K and *τ*_0_ = 2.9(2) × 10^−4^ s (Figure 5c). The very low *U*_eff_ value indicates that the QTM mechanism may work in this case. As for Yb@Y-sq (Figure 3a), no relaxation was recorded without any DC field at all, possibly ascribable to a QTM mechanism. Only when a DC field was applied, meaningful relaxation emerged. Thus, Yb@Y-sq can be regarded as a so-called field-induced SIM. A clear matrix dependence was observed between Yb@Y-sq and Yb@Lu-sq. The DC magnetic measurements also showed no hysteresis using our SQUID apparatus. The *M-H* curves on Yb@Y-sq and Yb@Lu-sq are displayed in Figure S2d.

The Cole–Cole plot [1,40] did not trace a simple semicircle for Dy@Lu-sq (Figure S8, Table S2). The ellipticities (the Cole-Cole parameters, *α*’s) of the plot measured at 3 K were determined as 0.43(3) and 0.30(8) at *H*_DC_ = 0 and 1000 Oe, respectively. The plots of the data at 11 K hardly exhibited any semicircles. However, the Cole-Cole plots for Dy@Y-sq at 3 and 12 K show simple semicircles for both *H*_DC_ = 0 and 1000 Oe. The fitting results indicate that *α* was determined as range from 0.10(2) to 0.534(7). A small *α* value indicates a narrow distribution of the relaxation times in a relatively high-temperature region. No significant matrix dependence was observed in the Dy-sq diluted samples for Arrhenius plots, but the relaxation process of Dy@Lu-sq would be more complex than Dy@Y-sq according to the Cole-Cole plot. In addition, Dy-sq derivatives often showed no simple relaxation process because Dy ions have strong anisotropy; in fact, a second relaxation process may be operative [41].

The diamagnetically diluted Yb-sq samples exhibited a simple semicircle in all cases (Figure S9, Table S2). The fitting results show *α* = 0.027(7) for Yb@Y-sq at 5.5 K and *α* = 0.06(3) for Yb@Lu-sq at 5 K at *H*_DC_ = 400 Oe. The *α* value was found to be 0.324(5) at 3 K for Yb@Lu-sq at *H*_DC_ = 0 Oe. The value is consistent with the results from the generalized Debye model treatment, where *α* ranged from 0.280(10) to 0.312(8) (Table S1). These values are relatively larger than those recorded at *H*_DC_ = 400 Oe, probably due to the contribution of tunneling pathways. As described in the Introduction, Yb^3+^ is a less studied member in RE ions for the development of SIMs [9,10]. This work confirmed the intrinsic SIM behavior from Yb@Lu-sq, though the QTM process disturbs the relaxation retardation.

## 3. Discussion

A few relaxation processes shortcut the magnetization reversal barrier in a low-temperature region, reducing *U*_eff_. To develop RE-based SIMs, the QTM process should be suppressed [42]. The crystal field symmetry of ideal *D*_4d_, *D*_5h_, *D*_6h_, and *C*_∞v_ are promising, because the mixed *m_J_* levels are regulated by vanishing off-diagonal crystal field parameters, effectively preventing the undesired QTM [43]. The *D*_4d_ symmetry is very frequently encountered among various coordination geometries. Along these lines, the *D*_4*d*_ crystal field found of the RE-sq family (Figure 1) enhances the axial anisotropy and maintains higher symmetry. All the crystals of RE-sq have CShM = 0.290 − 0.492 [12] with respect to SAPR-8 [37]. The approximate fourfold axis directions are nearly parallel in a crystal, possibly directing the local anisotropy axis nearly parallel. This structural feature seems to be another advantage for slowing magnetization reversal [38,39], in particular for the undiluted systems.

Ishikawa et al. reported the pioneering work on the SIM behavior from the oblate Tb^3+^ salt [Tb(pc)_2_]^−^ (pc = phthalocyanate) having an axially elongated SAPR structure [13,14]. On the other hand, Coronado et al. reported that the prolate Yb^3+^ salt [Yb(W_5_O_18_)_2_]^9−^ having a severely compressed SAPR behaved as an SIM [11,44]. Very interestingly, the Tb^3+^ counterpart, [Tb(W_5_O_18_)_2_]^9−^, did not behave [11]. Specifically, the nature of axial elongation and compression in *D*_4d_ structures plays a vital role in determining the magnetic anisotropy [45], and, consequently, the performance of SIMs. The moderate compression structure of the present RE-sq family seems to be beneficial for both Dy- and Yb-based SIM behavior.

Applying a DC bias field removes the ground-state degeneracy [17], and it is particularly effective when Kramers ions like Dy^3+^ and Yb^3+^ are chosen as spin sources [46]. These features are clearly observed in Figure 2 and Figure 3. Especially for the Dy derivatives, an upsurge of *χ*_AC_′ was recorded near the base temperature, 2 K (Figure 2a,c), ascribed to QTM. In sharp contrast, these *χ*_AC_′ profiles disappeared in Figure 2b,d. Another frequency-dependent *χ*_AC_′ hump was recorded around 8–14 K, reasonably assigned to an Orbach relaxation with overlapping Raman relaxation. It is unsurprising that the *U*_eff_ values did not change so drastically; 139(5) vs. 159(3) K for Dy@Y-sq (Figure 4a,b) and 135(8) vs. 163(6) K for Dy@Lu-sq (Figure 4c,d). As for the Yb derivatives, a *χ*_AC_′ upsurge similar to that of the Dy derivatives is observed in Figure 3c, whereas it is absent in Figure 3d. When combining the analysis result using the Arrhenius plot (Figure 5c), the relaxation is reasonably ascribed to QTM. The *U*_eff_ values are 61.2(14) and 62.5(16) K for Yb@Y-sq and Yb@Lu-sq, respectively, only slightly improved from that of undiluted Yb-sq (57 K) [12], which is again unsurprising. The broad *χ*_AC_″ peak in 4–7 K recorded for Yb-sq (Figure 3f) is essentially due to the Orbach and Raman relaxation.

The QTM process is mediated by transverse anisotropy, dipolar, exchange, or hyperfine interactions, which also reduces *U*_eff_ [47,48,49,50]. In some cases, slow relaxation of intermolecular origin has also been pointed out [51]. Actually, magnetic dilution technique clarified the hidden relaxation factor in Dy-sq and Yb-sq, as clearly shown in Figure 2a,c,e and Figure 3c,e, respectively. In the present case, magnetic dilution and applying *H*_DC_ are quite effective. Furthermore, the similarity has been pointed out between the two *χ*_AC_ vs. *T* profiles given from the experiments of magnetic dilution and applying *H*_DC_ [41]. It is because both techniques lead to the reduction in QTM in common. In the present study, we can see similar effects on the suppression of the QTM by magnetic dilution and applying a field (Figure 2a,c,e). As for the Yb case, however, the relaxation behaviors are different between the two techniques (Figure 3a,c,e). Very interestingly, the matrix or host dependence was found in the QTM of Yb@Lu-sq when compared with practically no relaxation behavior for Yb@Y-sq. To our knowledge, such a matrix dependence has rarely been reported. The matrix affects not only the QTM but also the Orbach and Raman relaxation.

A possibility of dipolar interaction with respect to the nuclear spin should be noted briefly [52]. The nuclear spins are *I* = 1/2 and 7/2 for ^89^Y (natural abundance 100%) and ^175^Lu (97.4%), respectively; these nuclei have requirement for hyperfine interaction. The sq ions intervene the metal ions in the present compounds. The inter-ion distances (*r*) between the nearest neighboring RE ions are 6.4110(7) Å for Y…Y and 6.3670(4) Å for Lu…Lu, which are substitutes for those of Dy…Y or Yb…Y and Dy…Lu or Yb…Lu, respectively. Since nuclear relaxation dependence might obey the 1/*r*^3^ rule, where *r* is the distance from electron to nucleus [53], this factor seems to be too small to explain the observable difference in the relaxation. In the experimental results, the absence of slow relaxation in the Y matrix is obvious, compared with that of the Lu matrix.

It may be related to the vibration exchange between the coordination structures around Lu and Yb ions in a crystal lattice. According to the hard-soft acid-base principle, Y and Lu ions are classified as hard and soft ions, respectively. The bonds between lanthanide and oxygen atoms are relatively electrostatic in Y complexes whereas relatively covalent in Lu complexes. In addition, Lu ions are heavier than Y ions, leading to different lattice vibrations, namely phonon–spin coupling, which must be changed. It has been suggested that incorporating heavier substituting is an effective strategy for suppressing the spin–phonon coupling [49]. This notion agrees with our experimental results. In fact, the RE-O IR absorptions around 610–620 cm^−1^ changed, depending upon lanthanide ions; Y-sq: 611, Dy@Y-sq: 607, Yb@Y-sq; 610, Dy-sq: 610, Yb-sq: 619, Dy@Lu-sq: 640, Yb@Lu-sq: 622, and Lu-sq: 622 cm^−1^. The present trend is very consistent with those of the previous reports [54]. Diluted Yb-based materials significantly affect the *χ*_AC_″ behavior as the matrix dependence. On the other hand, Dy-based materials did not exhibit such phenomena due to their overwhelming magnetic anisotropy.

## 4. Materials and Methods

### 4.1. Preparation

Complex Y-sq was prepared according to the method for the known RE-sq with a slight modification [12,29]. Diluted samples, Dy@Y-sq, Dy@Lu-sq, Yb@Y-sq, and Yb@Lu-sq were prepared in a manner similar to that of undiluted RE-sq samples. The starting lanthanide acetate salts were mixed with a molar ratio of ca. 1/99 before the complexation reaction. The composition formulas of the products (Dy_0.01_Y_0.99_-sq etc.) were confirmed from the magnetic study. The following procedure is typical. An aqueous solution (20 mL) contain 268 mg (0.792 mmol) of Y(OAc)_3_•4H_2_O (Wako Chemicals, Osaka, Japan) and 3.3 mg (0.008 mmol) of Dy(OAc)_3_•4H_2_O (Wako Chemicals) and an aqueous solution (30 mL) containing 183 mg (1.6 mmol) of squaric acid (H_2_sq) (Sigma-Aldrich, St. Louis, MO, USA) were combined at room temperature. After the resultant clear solution was allowed to stand at room temperature for a week, tiny clear platelet crystals of Dy@Y-sq were deposited. The crystals were collected on a filter, washed with chilled water, and finally air-dried. The yield was 240.8 mg (0.365 mmol; 91%). The crystals without further purification were subjected to analytical, spectroscopic, crystallographic, and magnetic analyses.

Y-sq, [Y_2_(sq)_3_(H_2_O)_8_]*_n_*: Yield: 90%, M.p.: >300 °C. Anal. Calcd. for C_12_H_16_EY_2_O_20_: C, 21.96; H, 2.46%. Found: C, 21.61; H, 2.28%. IR (neat, ATR): 611, 665, 858, 1093, 1137, 1459, 1605, 3084, 3404 cm^−1^.

Dy@Y-sq, [Dy_0.02_Y_1.98_(sq)_3_(H_2_O)_8_]*_n_*: Yield: 91%, M.p.: >300 °C. Anal. Calcd. for C_12_H_16_Dy_0.02_Y_1.98_O_20_: C, 21.85; H, 2.45%. Found: C, 21.80; H, 2.23%. IR (neat, ATR): 607, 665, 858, 1093, 1137, 1456, 1604, 3077, 3405 cm^−1^.

Dy@Lu-sq, [Dy_0.02_Lu_1.98_(sq)_3_(H_2_O)_8_]*_n_*: Yield: 72%, M.p.: >300 °C. Anal. Calcd. for C_12_H_16_Dy_0.02_Lu_1.98_O_20_: C, 17.36; H, 1.95%. Found: C, 17.38; H, 1.77%. IR (neat, ATR): 640, 667, 742, 859, 1096, 1137, 1463, 1604, 3063, 3400 cm^−1^.

Yb@Y-sq, [Yb_0.02_Y_1.98_(sq)_3_(H_2_O)_8_]*_n_*: Yield: 88%, M.p.: >300 °C. Anal. Calcd. for C_12_H_16_Yb_0.02_Y_1.98_O_20_: C, 21.84; H, 2.45%. Found: C, 21.80; H, 2.16%. IR (neat, ATR): 610, 665, 858, 1093, 1137, 1459, 1604, 3080, 3399 cm^−1^.

Yb@Lu-sq, [Yb_0.02_Lu_1.98_(sq)_3_(H_2_O)_8_]*_n_*: Yield: 83%, M.p.: >300 °C. Anal. Calcd. for C_12_H_16_Yb_0.02_Lu_1.98_O_20_: C, 17.36; H, 1.95%. Found: C, 17.53; H, 1.66%. IR (neat, ATR): 622, 667, 741, 859, 1094, 1138, 1456, 1605, 3070, 3398 cm^−1^.

### 4.2. Structural Analysis

The powder X-ray diffraction (PXRD) patterns of RE-sq-type compounds were recorded on a Rigaku Ultima III diffractometer (Tokyo, Japan) using Cu Kα radiation (*λ* = 1.54178 Å) at room temperature. The single-crystal X-ray diffraction data on Y-sq were collected on a Rigaku XtaLAB Synergy R HyPix diffractometer with graphite monochromated Mo *K*α radiation (*λ* = 0.71073 Å). The *hkl* and intensity data were extracted in CrysAlisPro 1.171.43.105a [55]. The structure was directly solved and expanded using Fourier techniques in the Olex2 1.5 program [56]. The parameters were refined on Shelxl 2014/7 [57]. Hydrogen atoms were placed at calculated positions, and their parameters were treated as “riding”. A twin analysis was applied, giving the fraction ratio 0.708/0.292. Selected crystallographic parameters are as follows. C_12_H_16_O_20_Y_2_, monoclinic, *Pc*, *a* = 11.9494(5) Å, *b* = 8.1543(4) Å, *c* = 10.0321(4) Å, *β* = 95.805(4)°, *V* = 972.51(8) Å^3^, *Z* = 2*, d*_calc_ = 2.247 g cm^−3^, *μ*(MoKα) = 6.046 mm^−1^, *R*(*F*) [*I* > 2*σ*(*I*)] = 0.0273, *R*_w_(*F*^2^) (all data) = 0.0614, G.O.F. = 1.030, and *T* = 297 K for 4365 unique reflections. CCDC reference number 2402342.

### 4.3. Magnetic Analysis

The DC magnetic susceptibilities of diamagnetically diluted RE-sq samples were measured on a Quantum Design MPMS-XL7 SQUID magnetometer (San Diego, CA, USA) with a static field of 500 Oe. The magnetic responses were corrected with diamagnetic blank data of the sample holder and the pure matrices Y-sq and Lu-sq measured separately. The diamagnetic contribution of the sample itself was estimated by Pascal’s constants [58]. FCM, ZFCM, and RM of RE-sq were measured on a Quantum Design MPMS-3 magnetometer. The AC magnetic susceptibilities on diluted samples as well as undiluted Dy-sq and Yb-sq were recorded on a Quantum Design MPMS-3 magnetometer equipped with an AC SQUID option. The measurements in the AC frequency range of 10 to 10 kHz required a Quantum Design PPMS apparatus with an AC/DC magnetization option.

## 5. Conclusions

Thanks to the isostructural nature, we explored the effects of diamagnetic dilution on the SIM behavior in the 2D polymeric RE-sq series. Diluted Dy compounds, Dy@Y-sq and Dy@Lu-sq, showed AC magnetic susceptibility peaks in both systems. The Orbach and Raman mechanisms are assumed in the Arrhenius plots of the data at *H*_DC_ = 0 Oe. The effect of the magnetic dilution is obvious, because undiluted Dy-sq showed no frequency-dependent AC susceptibility under the same conditions. No matrix dependence was observed. On the other hand, Yb@Y-sq and Yb@Lu-sq behaved different; Yb@Y-sq can be regarded as a field-induced SIM, while Yb@Lu-sq showed a relaxation even at *H*_DC_ = 0 Oe, ascribable to a QTM mechanism. The results on Yb@Y-sq and Yb@Lu-sq demonstrated the matrix dependence.

This study offers a comprehensive examination of the underlying relaxation mechanisms in SIMs. Though the dilution method is technically very easy, relaxation is a facile and versatile tool for modification of SIM characteristics. This research not only contributes to the fundamental understanding of SIMs but also provides practical insights for the design of next-generation magnetic materials with tailored properties.

## Data Availability

Data are contained within the article and Supplementary Materials. CCDC deposition number 2402342 contains the supplementary crystallographic data for Y-sq. These data are provided free of charge by the Cambridge Crystallographic Data Centre at www.ccdc.cam.ac.uk/structures (accessed on 10 January 2025).

## Supplementary Materials

The following supporting information can be downloaded at: https://www.mdpi.com/article/10.3390/molecules30020356/s1, Figure S1: DC magnetic measurements for Y-sq and Lu-sq; Figure S2: DC magnetic measurements for Dy@Y-sq, Dy@Lu-sq, Yb@Y-sq, and Yb@Lu-sq; Figure S3: DC magnetization for Dy@Lu-sq using a ^3^He refrigerator; Figure S4: FCM, ZFCM, and RM measurements for Dy@Y-sq and Dy@Lu-sq; Figure S5: Field dependence of *χ*_AC_ for Dy@Y-sq and Dy@Lu-sq; Figure S6: AC susceptibility vs *T* plot at *H*_DC_ = 3000 Oe for Dy-sq; Figure S7: AC magnetic susceptibilities and Cole-Cole plot for Yb@Lu-sq based on the generalized Debye model; Figure S8: Cole-Cole plots of Dy@Y-sq and Dy@Lu-sq; Figure S9: Cole-Cole plots of Yb@Y-sq and Yb@Lu-sq; Table S1: Parameters for the generalized Debye model for Yb@Lu-sq; Table S2: Parameters for the Cole-Cole analysis for Dy@Y-sq, Dy@Lu-sq, Yb@Y-sq, and Yb@Lu-sq.
