# Supplementary material for: Magnetically Diluted Dy3+ and Yb3+ Squarates Showing Relaxation Tuning and Matrix Dependence
Source: Molecules. 2025 Jan 16;30(2):356. doi: 10.3390/molecules30020356 (PMC11767464; doi:10.3390/molecules30020356)
Supplement: Supplementary file 1 [file molecules-30-00356-s001.zip › molecules-3389717-supplementary.pdf]

Supporting Information For:

Magnetically Diluted Dy^{3+} and Yb^{3+} Squarates Showing Relaxation Tuning and Matrix Dependence

Rina Takano and Takayuki Ishida *

Department of Engineering Science, The University of Electro-Communications, Chofu 182-8585, Tokyo, Japan

* Correspondence: takayuki.ishida@uec.ac.jp; Tel.: +81-42-443-5490; Fax: +81-42-443-5501

Contents

Contents		Page
**Figure S1.**	DC magnetic measurements for Y-sq and Lu-sq	p. S2
**Figure S2.**	DC magnetic measurements for Dy@Y-sq, Dy@Lu-sq, Yb@Y-sq, and Yb@Lu-sq	p. S2
**Figure S3.**	DC magnetization for Dy@Lu-sq using a ^{3}He refrigerator	p. S3
**Figure S4.**	FCM, ZFCM, and RM measurements for Dy@Y-sq and Dy@Lu-sq	p. S3
**Figure S5.**	Field dependence of χ_{AC} of Dy@Y-sq and Dy@Lu-sq	p. S3
**Figure S6.**	AC susceptibility vs T plot at H_{DC} = 3000 Oe for Dy-sq	p. S4
**Table S1.**	Parameters for the generalized Debye model for Yb@Lu-sq	p. S4
**Figure S7.**	AC magnetic susceptibilities and Cole-Cole plot for Yb@Lu-sq based on the generalized Debye model	p. S5
**Table S2.**	Parameters for the Cole-Cole analysis for Dy@Y-sq, Dy@Lu-sq, Yb@Y-sq, and Yb@Lu-sq	p. S5
**Figure S8.**	Cole-Cole plots of Dy@Y-sq and Dy@Lu-sq	p. S6
**Figure S9.**	Cole-Cole plots of Yb@Y-sq and Yb@Lu-sq	p. S7
**References**		p. S8

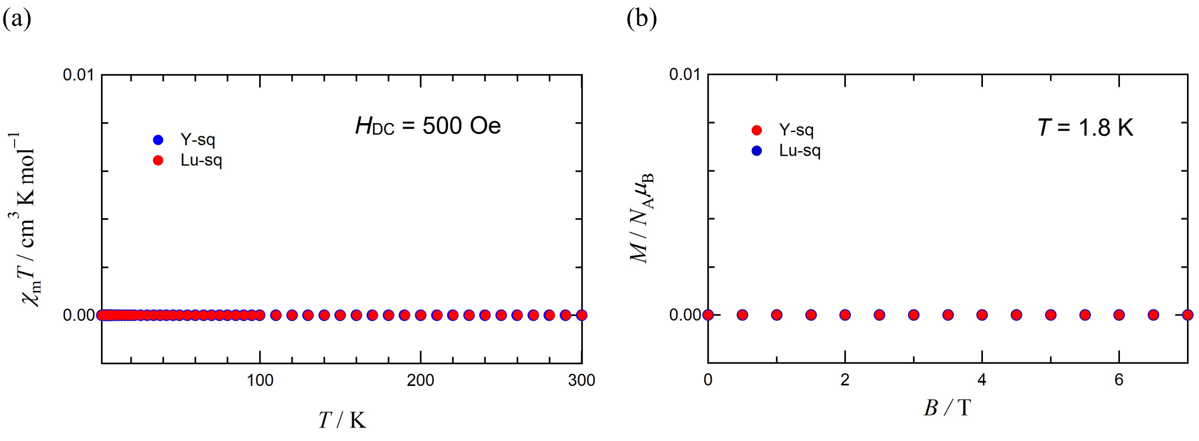

Figure S1. (a) DC magnetic susceptibilities and (b) magnetizations, calculated per the formula $[RE_2(sq)_3(H_2O)_8]$ (RE = Y, Lu).

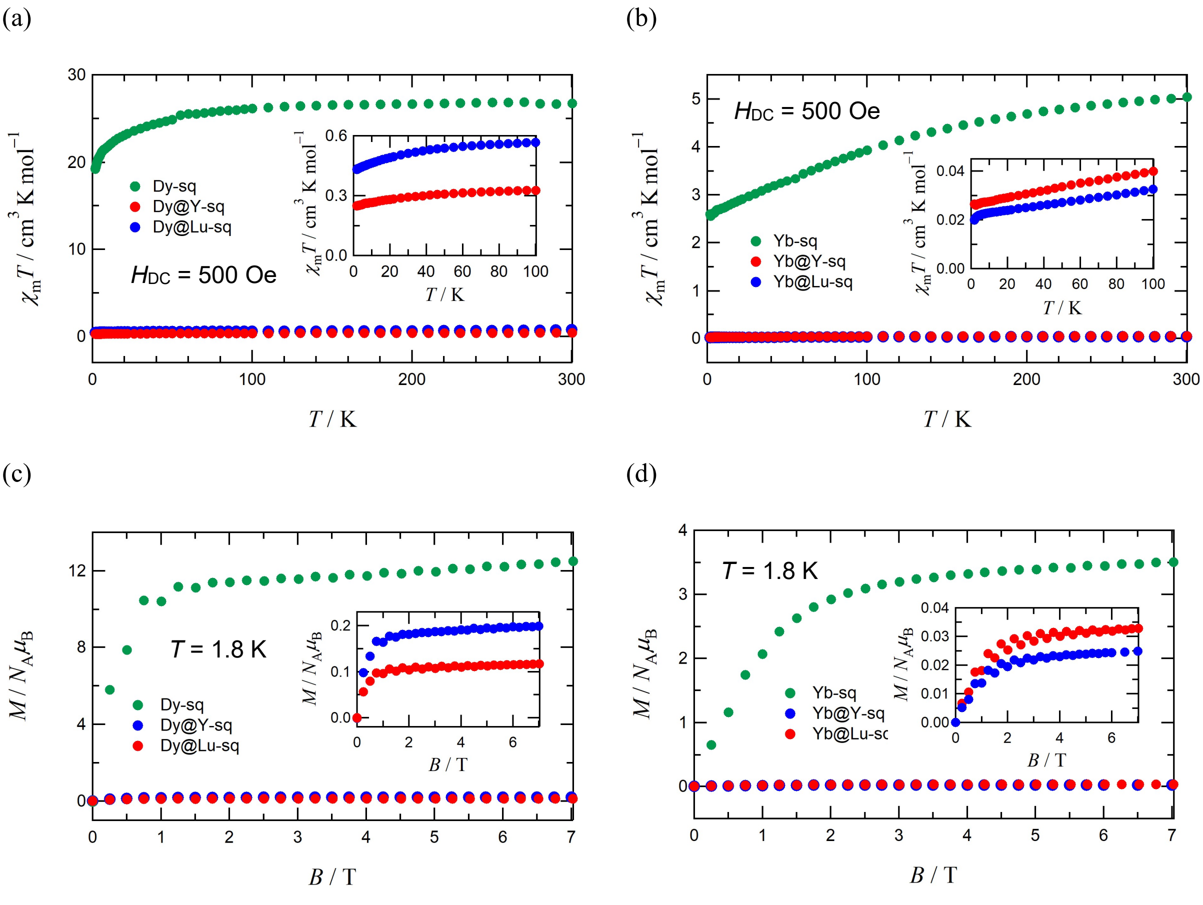

Figure S2. DC magnetic susceptibilities for (a) Dy@Y-sq and Dy@Lu-sq and (b) Yb@Y-sq and Yb@Lu-sq. Magnetizations for (c) Dy@Y-sq and Dy@Lu-sq and (d) Yb@Y-sq and Yb@Lu-sq. The diamagnetic contribution from matrices Y-sq or Lu-sq (Figure S1) were subtracted from the data of the diluted samples. The susceptibilities and magnetizations are calculated per the formula $[RE_2(sq)_3(H_2O)_8]$.

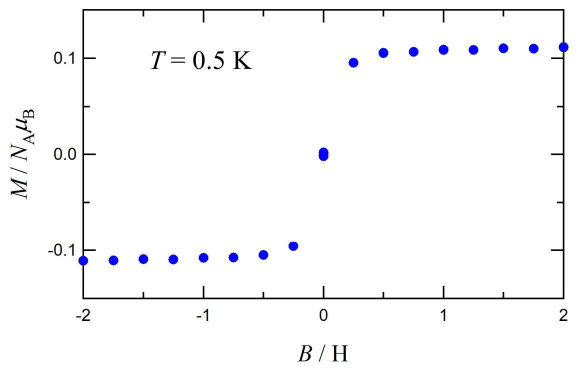

Figure S3. DC magnetization for Dy@Lu-sq, measured at ca. 0.5 K using a [3]He refrigerator. Magnetization is calculated per the formula [$Dy_{0.02}Lu_{1.98}(sq)_3(H_2O)_8$].

(a) (b)

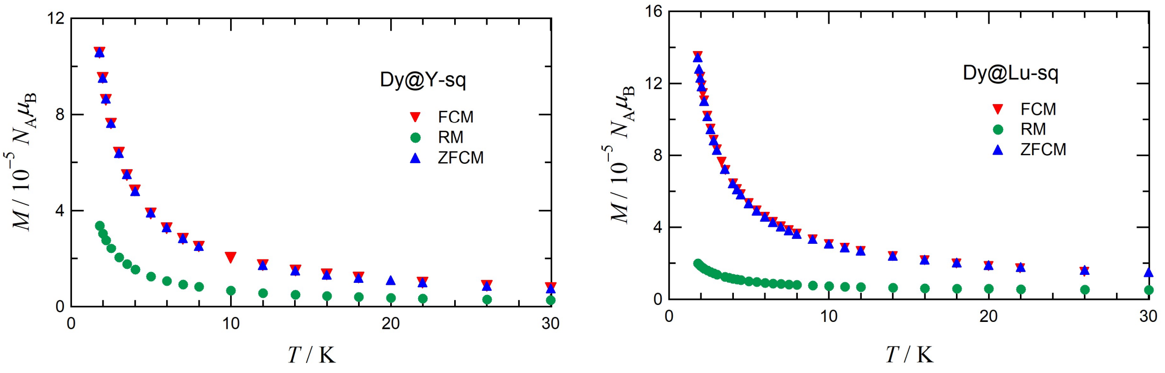

Figure S4. Field-cooled magnetization (FCM), zero-field-cooled magnetization (ZFCM), and remnant magnetization (RM) for (a) Dy@Y-sq and (b) Dy@Lu-sq. FCM and ZFCM measurements were performed at H_{DC} = 3 Oe. Magnetizations are calculated per the formula [$Dy_{0.02}RE_{1.98}(sq)_3(H_2O)_8$] (RE = Y, Lu).

(a) (b)

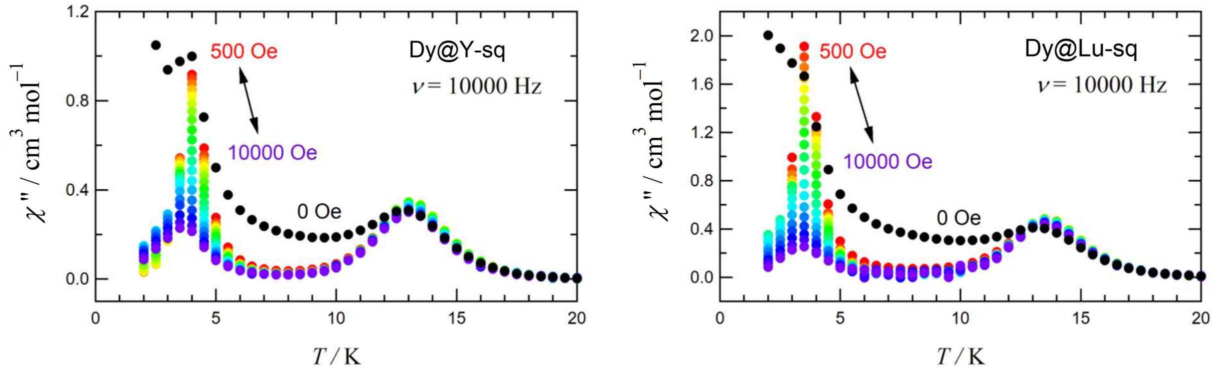

Figure S5. Field dependence of AC out-of-phase magnetic susceptibilities for (a) Dy@Y-sq and (b) Dy@Lu-sq with ac field frequency ν = 10 kHz. The susceptibilities are converted per the undiluted composition formula [$Dy_2(sq)_3(H_2O)_8$].

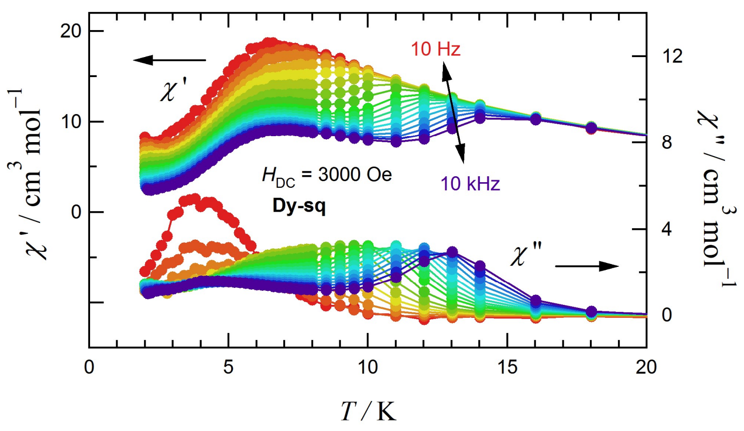

Figure S6. AC magnetic susceptibilities with H_{DC} = 3000 Oe for Dy-sq. The susceptibilities are calculated per the formula $[Dy_2(sq)_3(H_2O)_8]$.

Table S1. Parameters of the generalized Debye model analysis for Yb@Lu-sq without any DC field.[a]

Temp.	χ_T / cm^3 mol^{-1}	χ_S / cm^3 mol^{-1}	α	τ / 10^{-4} s
2.0	0.878(3)	0.271(8)	0.280(10)	4.52(12)
2.2	0.796(3)	0.229(7)	0.282(9)	4.20(12)
2.4	0.732(2)	0.191(7)	0.310(9)	3.87(12)
2.6	0.673(2)	0.171(6)	0.305(9)	3.79(10)
2.8	0.6227(17)	0.182(5)	0.280(8)	4.15(10)
3.0	0.5826(15)	0.156(5)	0.296(8)	3.92(9)
3.2	0.5458(15)	0.130(5)	0.312(8)	3.55(10)
3.4	0.5139(17)	0.130(7)	0.305(11)	3.69(15)
3.6	0.4876(14)	0.123(6)	0.309(9)	3.79(14)
3.8	0.4612(12)	0.110(4)	0.311(8)	3.57(9)

[a] Generalized Debye model fitting was performed with eqs. S1 and S2 [S1].

$$\chi'_{(\omega)} = \chi_S + (\chi_T - \chi_S)\frac{1 + (\omega\tau)^{(1-\alpha)}\sin\left(\frac{\pi\alpha}{2}\right)}{1 + 2(\omega\tau)^{(1-\alpha)}\sin\left(\frac{\pi\alpha}{2}\right) + (\omega\tau)^{2(1-\alpha)}} \qquad \text{(eq. S1)}$$

$$\chi''_{(\omega)} = \chi_S + (\chi_T - \chi_S)\frac{1 + (\omega\tau)^{(1-\alpha)}\cos\left(\frac{\pi\alpha}{2}\right)}{1 + 2(\omega\tau)^{(1-\alpha)}\sin\left(\frac{\pi\alpha}{2}\right) + (\omega\tau)^{2(1-\alpha)}} \qquad \text{(eq. S2)}$$

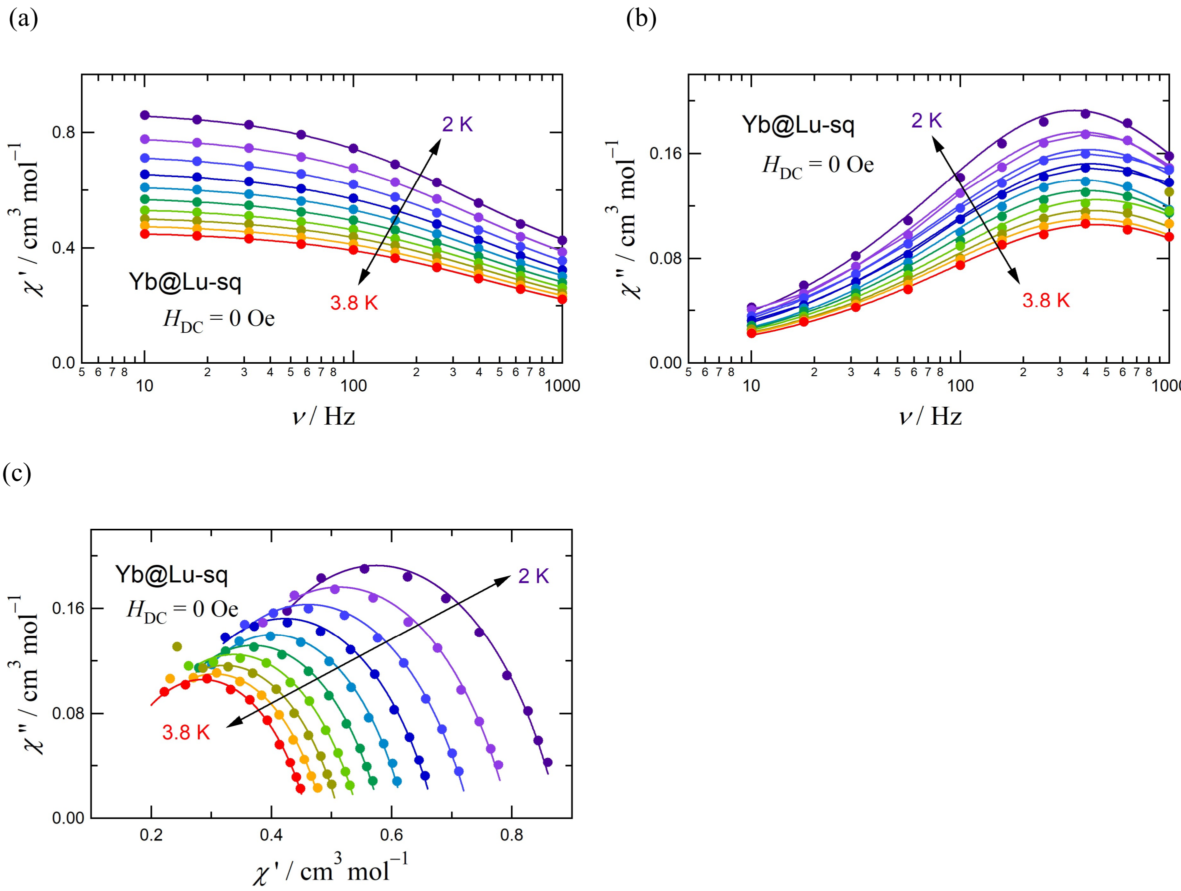

Figure S7. AC magnetic susceptibilities (a) χ_{AC}' and (b) χ_{AC}'' for Yb@Lu-sq as a function of frequency, and (c) the Cole-Cole plot. Solid lines stand for the generalized Debye model with optimized parameters summarized in Table S2. The susceptibilities are converted per the undiluted composition formula [$Yb_2(sq)_3(H_2O)_8$].

Table S2. Parameters for the Cole-Cole analysis.[a]

	H / Oe	T / K	χ_T / cm^3 mol^{-1}	χ_S / cm^3 mol^{-1}	α
Dy@Y-sq	0	3	6.82(4)	0.48(5)	0.534(7)
		12	1.871(9)	1.06(7)	0.15(6)
	1000	3	3.50(4)	0.232(6)	0.232(8)
		12	1.891(3)	0.99(2)	0.10(2)
Dy@Lu-sq	0	3	5.70(8)	1.49(17)	0.43(3)
		11	1.70(6)	1.17(15)	0.2(3)
	1000	3	3.61(18)	0.04(18)	0.30(8)
		11	1.73(4)	1.07(2)	0.05(9)
Yb@Y-sq	400	5.5	0.3932(6)	0.0305(11)	0.027(7)
Yb@Lu-sq	0	3	0.5865(7)	0.143(3)	0.324(5)
	400	5	0.531(10)	0.040(4)	0.06(3)

[a] The fitting was performed with eq. S3 [S1].

$$\chi_{(\omega)} = \chi_S + \frac{\chi_T - \chi_S}{1 + (i\omega\tau)^{1-\alpha}} \qquad \text{(eq. S3)}$$

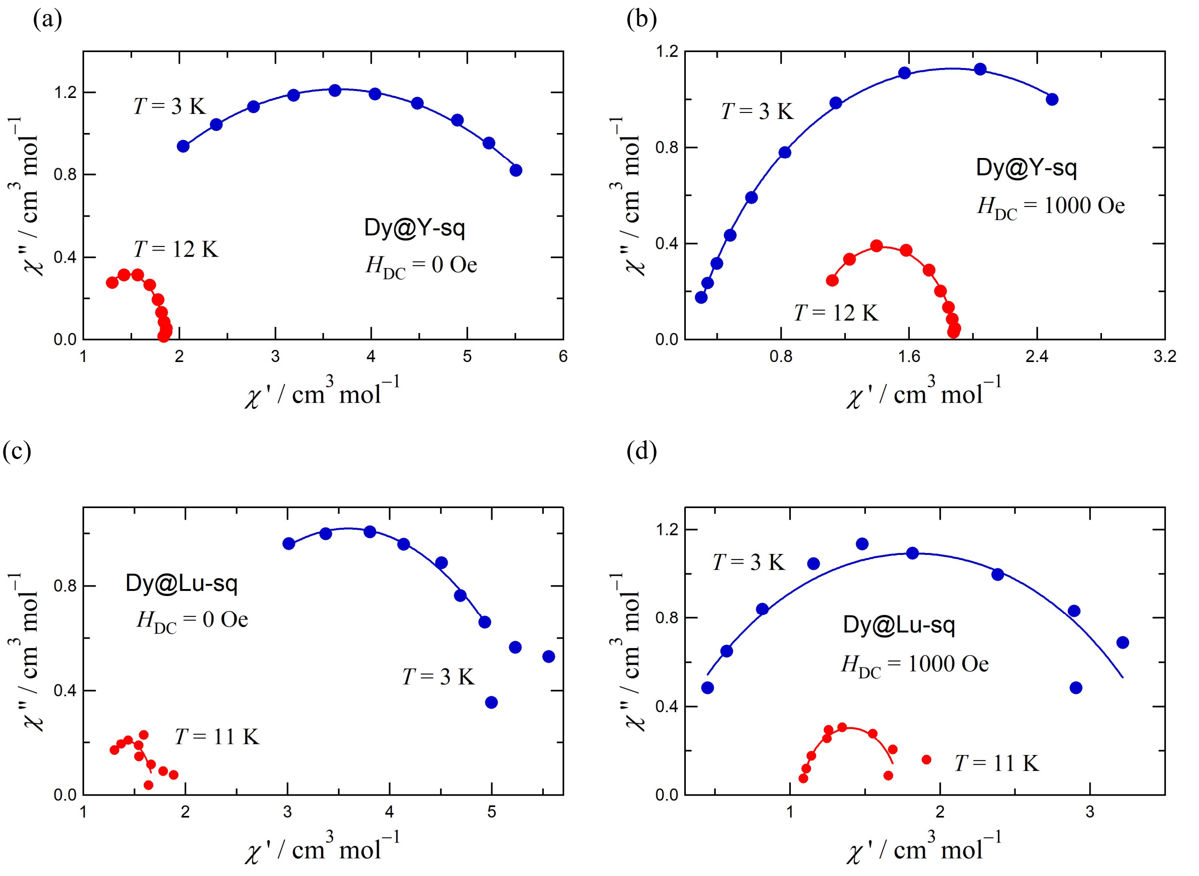

Figure S8. Cole-Cole plots for Dy@Y-sq at H_{DC} = 0 Oe (a), Dy@Y-sq at H_{DC} = 1000 Oe (b), Dy@Lu-sq at H_{DC} = 0 Oe (c), and Dy@Lu-sq at H_{DC} = 1000 Oe (d). The data are given from Figure 2. The blue and red curves represent the fitting results at 5 K and 12 or 11 K, respectively. Optimized parameters are listed in Table S2.

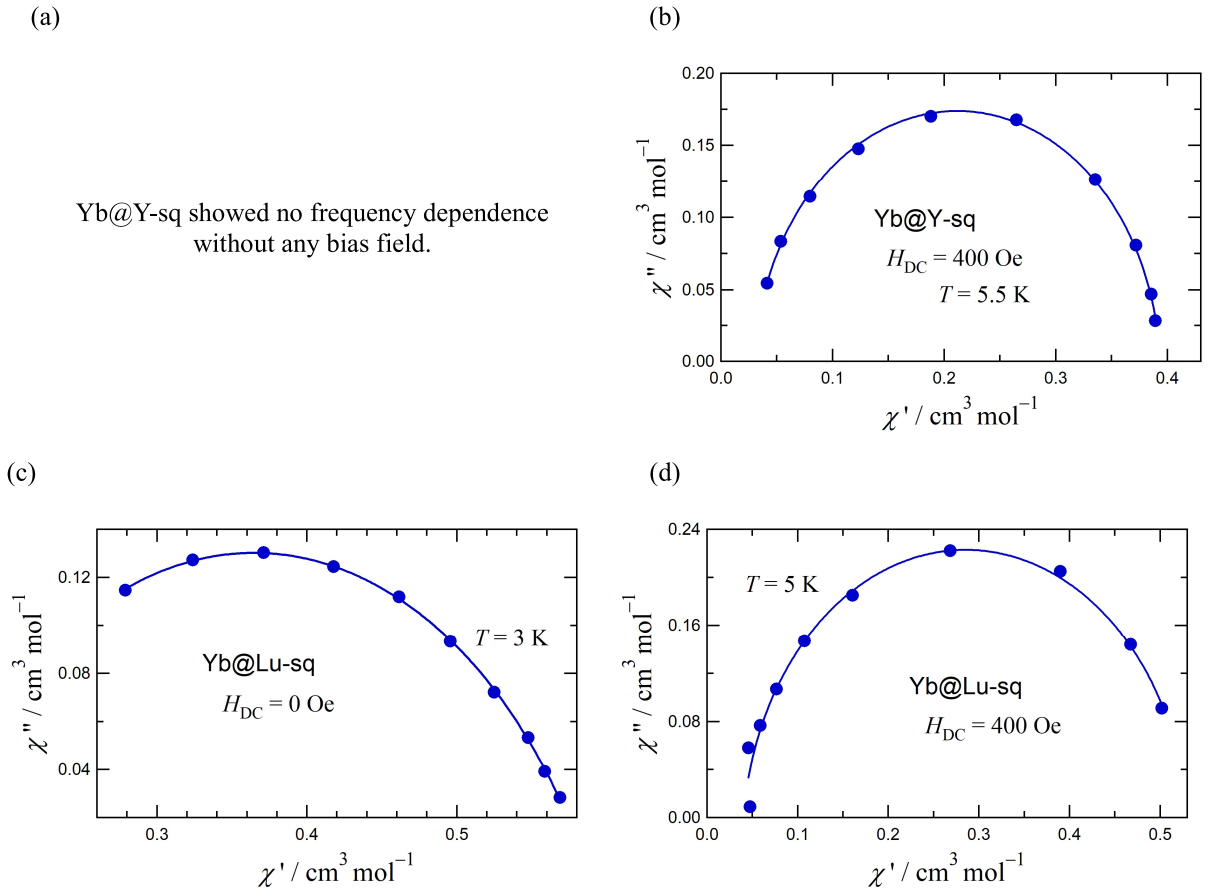

Figure S9. Cole-Cole plots for Yb@Y-sq at H_{DC} = 0 Oe (a), Yb@Y-sq at H_{DC} = 400 Oe (b), Yb@Lu-sq at H_{DC} = 0 Oe (c), and Yb@Lu-sq at H_{DC} = 400 Oe (d). The data are given from Figure 3. The blue curve represents the fitting results. Optimized parameters are listed in Table S2.

References

[S1] Gatteschi, D.; Sessoli, R.; Villain, J. *Molecular Nanomagnets*; Oxford University Press: Oxford, UK, 2006.
